# Inherited Chromosomally Integrated Human Herpesvirus 6: Laboratory and Clinical Features

**DOI:** 10.3390/microorganisms11030548

**Published:** 2023-02-21

**Authors:** Liliana Gabrielli, Alice Balboni, Eva Caterina Borgatti, Giulio Virgili, Evangelia Petrisli, Alessia Cantiani, Matteo Pavoni, Federico Baiesi Pillastrini, Simona Venturoli, Giulia Piccirilli, Tiziana Lazzarotto

**Affiliations:** 1 Microbiology Unit, IRCCS Azienda Ospedaliero-Universitaria di Bologna, 40138 Bologna, Italy; 2 Department of Medical and Surgical Sciences, University of Bologna, 40138 Bologna, Italy; 3 Infective Disease Unit, IRCCS Azienda Ospedaliero-Universitaria di Bologna, 40138 Bologna, Italy

**Keywords:** HHV-6, chromosomal integration, telomeres, hair follicles

## Abstract

Inherited chromosomally integrated human herpesvirus 6 (iciHHV-6) is a condition in which the complete HHV-6 genome is integrated into the chromosomes of the host germ cell and is vertically transmitted. The aims of this study were to identify iciHHV-6 prevalence in hospitalized patients and clinical features in individuals carrying this integration. HHV-6 PCR on hair follicles was used to confirm iciHHV-6 status when the blood viral load was more than 5 Log_10_ copies/mL. From January 2012 to June 2022, HHV-6 DNAemia was investigated in 2019 patients. In particular, 49 had a viral load higher than 6 Log_10_ copies/mL and HHV-6 DNA in hair follicles was positive. A viral load between 5.0 and 5.9 Log_10_ copies/mL was observed in 10 patients: 6 infants with acute HHV-6 infection and 4 patients with leukopenia and HHV-6 integration. Therefore, the iciHHV-6 prevalence in our population was 2.6% (53/2019). Adult patients with integration presented hematological (24%), autoimmune (11%), autoimmune neurological (19%), not-autoimmune neurological (22%), and other diseases (19%), whereas 5% had no clinically relevant disease. Although in our study population a high percentage of iciHHV-6 adult hospitalized patients presented a specific pathology, it is still unknown whether the integration is responsible for, or contributes to, the disease development.

## 1. Introduction

Human Herpesvirus 6 (HHV-6) is a Roseolovirus that comprises two distinct viruses, HHV-6A and HHV-6B [1,2].

HHV-6B infects nearly 100% of human beings [3], typically before the age of three and is the causative agent in *exanthema subitum* (also known as roseola infantum) [4,5,6], a childhood disease characterized by high fever, diarrhea, and a mild skin rash [7]. This initial infection can also cause febrile seizures and febrile status epilepticus [7,8].

Little is known about the prevalence of HHV-6A or how it is acquired [5,9]. It is generally believed that primary HHV-6A infection is acquired later than HHV-6B, through asymptomatic infection [6]. Recent findings suggest that high HHV-6A antibody levels are associated with increased multiple sclerosis risk and act synergistically with common environmental/lifestyle risk factors [10].

Like other herpesviruses, HHV-6 establishes latency [6,11], and reactivation can cause disease in severely immunocompromised hosts [9,12]. Multiple studies have described an association between reactivation of latent HHV-6 and encephalitis, graft rejection, acute Graft-versus-Host-Disease (GvHD), and bone marrow suppression in recipients of hematopoietic stem cell transplantation (HSCT) [13,14,15,16,17,18].

Unlike other herpesviruses, HHV-6 species establish latency through integration of the HHV-6 genome into the chromosomal telomeres [19,20,21,22], similarly to Marek’s disease virus, whose genomic integration is tightly associated with chicken lymphoma development [23]. HHV-6 integration occurs in a small proportion of somatic cells [7], including monocytes, macrophages, T-cells, and bone marrow progenitor cells [7,24,25]. The integration process is facilitated by telomeric repeats present at the ends of the viral DNA [26]. This allows maintaining the viral genome in latently infected cells, while no circular episome was observed [7].

Apart from integration into somatic cells, HHV-6A and B integration into the chromosomes of germ cells is possible in a selected number of patients and leads to offspring that carry a copy of the viral genome in every nucleated cell of the body [11,21,27,28,29]. This condition is referred as inherited chromosomally integrated HHV-6 (iciHHV-6) and occurs in approximately 1% of the human population [21,28,30,31,32,33], which corresponds to nearly 70 million people [19]. It remains unknown if integration in the germ cells occurs during primary infection or reactivation [7].

Heritable integration might occur in sperm progenitor cells or in an ovum [7]. The inherited viral genome is passed on to subsequent generations according to Mendelian laws [27]: half of the gametes harbor the virus resulting in about 50% of descendants carrying the virus in their germline [21]. iciHHV-6 individuals harbor one copy of the viral genome in every nucleated cell which accounts for the persistent high viral load in whole blood [5,34]. HHV-6 levels in whole blood that exceed 5.5 Log_10_ copies/mL are strongly suggestive of iciHHV-6 [5]. Rarely, in the absence of iciHHV-6, patients with GvHD or drug induced hypersensitivity syndrome can have transient viral load above 5.5 Log_10_ copies/mL [5,35].

In addition to genetic transmission, populations of cells harboring iciHHV-6 may be horizontally transmitted via allogenic HSCT [5] and via solid organ transplantation [36,37].

The prevalence of iciHHV-6 was found to be higher in hospitalized individuals (2.9 and 3.3%) than in blood donors (0.8 and 1.5%), possibly suggesting an impact on health, although not associated with any particular disease [20,38]. In fact, the consequences of having iciHHV-6A/B are not well defined [5,39], although there are evidences of association to a higher risk of developing angina pectoris [9]. Furthermore, iciHHV-6 subjects have shorter telomeres, which may explain, at least in part, how iciHHV-6 may contribute to the development of angina [9].

The aims of this study are to evaluate the iciHHV-6 frequency in hospitalized patients in Bologna, Italy, and the clinical features in iciHHV-6 patients. We used a cut-off of 5 Log_10_ HHV-6 copies/mL in whole blood and testing of hair follicles to confirm or exclude iciHHV-6 status. The occurrence of hematological, autoimmune, autoimmune and not-autoimmune neurological and other diseases in iciHHV-6 patients was investigated.

## 2. Materials and Methods

### 2.1. Patients Studied

Patients admitted to IRCCS St. Orsola Polyclinic, Maggiore Hospital, and Bellaria Hospital in Bologna investigated for HHV-6 DNA in whole blood from January 2012 to June 2022 were studied. When the HHV-6 DNA load in blood was greater than or equal to 5 Log_10_ copies/mL, the HHV-6 PCR test on hair follicles was used to confirm or exclude iciHHV-6 status [38,40], according to our protocol.

Clinical data and laboratory findings regarding iciHHV-6 patients were discussed in multidisciplinary team meetings. iciHHV-6 patients were divided in two age groups (<18 years and ≥18 years) and then classified according to their past and present medical history.

### 2.2. DNA Extraction and HHV-6 Detection Using Real-Time PCR

HHV-6 PCR was performed on EDTA-anticoagulated whole blood samples by using the HHV-6 ELITe MGB^®^ kit (ELITech Group, Turin, Italy), a commercial quantitative real-time PCR assay with the limit of detection (LOD) equal to 10 copies of target DNA per amplification reaction. In each well, two amplification reactions were performed: one for the ORF13R region, belonging to the U67 gene of HHV6, and one human beta globin gene (internal control). The test is not able to discriminate between HHV-6 A and B. The assay was used in association with different extractions and amplification platforms, as follows.

Until May 2018, DNA extraction was performed with the QIAsymphony SP instrument (Qiagen, Hilden, Germany) and the quantification of HHV-6 was performed by using HHV-6 ELITe MGB^®^ kit on the ABI Prism 7500 real-time PCR System (PE Applied Biosystem, Foster City, CA, USA), according to the assay manufacturer’s instructions. In brief: 0.5 mL of samples were extracted; the DNA was eluted in 100 µL, and 20 µL of DNA were used for amplification. The LOD and the lower limit of quantification (LLQ) of such method were 120 copies/mL, whereas the upper limit of quantification (ULQ) of the assay was 12,000,000 copies/mL.

From June 2018, HHV-6 ELITe MGB^®^ Kit was used in association to the ELITeInGenius^®^ instrument, an integrated platform for fully automated nucleic acids extraction and real-time PCR amplification. The extraction was performed with ELITe InGenius SP 200 (ELITechGroup S.p.A., Torino, Italy), a set of dedicated reagents for nucleic acid extraction from different matrices. The samples were processed using a pre-set protocol: 0.2 mL of samples were extracted, the DNA was eluted in 100 µL, and 20 µL of DNA were used for amplification. The LOD and LLQ of this method were 131 copies/mL, and ULQ was 17,857,143 copies/mL.

Considering hair follicles testing, at least 4 hair follicles were collected for each patient. The samples were added to 180 µL of tissue lysis buffer (ATL buffer, Qiagen) and 20 µL of protease (Proteinase K solution, Qiagen, Germany), incubated overnight at 56 °C and then for one hour at 90 °C. Extraction of nucleic acids and real-time PCR amplification were performed using the same protocol of whole blood and qualitative results were expressed as positive or negative. A sample was considered truly negative when ORF13R was not amplified (Ct undetermined for HHV-6) and beta globin gene was detected with Ct ≤ 35. The human beta globin region was used as an endogenous internal control for sufficient specimen cellularity and for extraction and amplification steps.

### 2.3. Statistical Analysis

The Fisher’s exact test was used to compare the distribution of iciHHV-6 between male and female subjects and of patients without underlying medical conditions between the two age groups. *p* < 0.05 was considered statistically significant.

95% confidence intervals (CI) analysis were computed. Analyses were performed using Statsoft Version 7.1 (StatSoft Europe GmbH, 22301 Hamburg, Germany) for Windows 10.

## 3. Results

From January 2012 to June 2022, HHV-6 DNA in whole blood was investigated in 2019 patients (age range: 0–92, mean age: 37) admitted to IRCCS St. Orsola Polyclinic, Maggiore Hospital, and Bellaria Hospital in Bologna, under clinicians’ requests.

Of the 2019 hospitalized patients, 1464 (72.5%) had HHV-6 DNA negative result in whole blood, whereas 555 (27.5%) patients had a positive result. HHV-6 positive patients were classified according to the viral load: 365 (66%) patients had a viral load <2 Log_10_ copies/mL (LLQ); 131 (23%) had a viral load between 2.0–5.0 Log_10_ copies/mL (range 2.5–4.5 Log10 copies/mL; IQR 3.4) and 59 (11%) had a viral load ≥5.0 Log10 copies/mL (range 5–7.2 Log10 copies/mL; IQR 6.4).

The 59 patients with a viral load ≥5.0 Log_10_ copies/mL were then tested by HHV-6 PCR on hair follicles. The results obtained are summarized in Table 1.

In particular, 49/59 (83%) patients had a viral load ≥6 Log_10_ copies/mL (range: 6.0–7.2) and all were positive for HHV-6 DNA in hair follicles, thus confirming the assumption of iciHHV-6.

The remaining 10 patients had a viral load between 5 and 5.9 Log_10_ copies/mL. Six patients were infants, 2 affected by hematologic malignancy, and 2 were transplant recipients.

The 6 infants (age: 10 days–2 years) had fever and rash clinically compatible with roseola infantum and the viral load was between 5.1 and 5.5 Log_10_ copies/mL. Their clinical condition was so critical as to require admission to hospital and further investigations. In particular, one infant was 2 years old and had had recent infection with severe acute respiratory syndrome coronavirus-2 and acute hepatitis. The others were between 10 to 19 days of life, two with neutropenia, two with febrile seizures and one with concomitant urinary tract infection. The acute HHV-6 infection was confirmed by the negative result of hair follicles and the progressive reduction of blood viral load without antiviral treatment.

Of the 2 patients with hematologic malignancy, one had acute myeloid leukemia, and presented with fever, rash and leukopenia (leukocytes: 730/µL) at the onset of the disease. HHV-6 viral load in the blood was 5 Log_10_ copies/mL. The suspected iciHHV-6 status was confirmed by the hair follicles’ positive PCR. The other hematologic malignancy patient had an acute lymphoblastic leukemia, and HHV-6 was investigated during screening exams prior to the chemotherapy administration. The blood viral load was 5.2 Log_10_ copies/mL associated with leukopenia (leukocytes: 330/µL), and the iciHHV-6 suspect was confirmed by the hair follicles’ positive PCR. After 14 days from the first investigation the HHV-6 load increased up to 6.0 Log_10_ copies/mL in correspondence with the increase in the number of leukocytes (leukocytes: 2760/µL).

**Figure 1:**
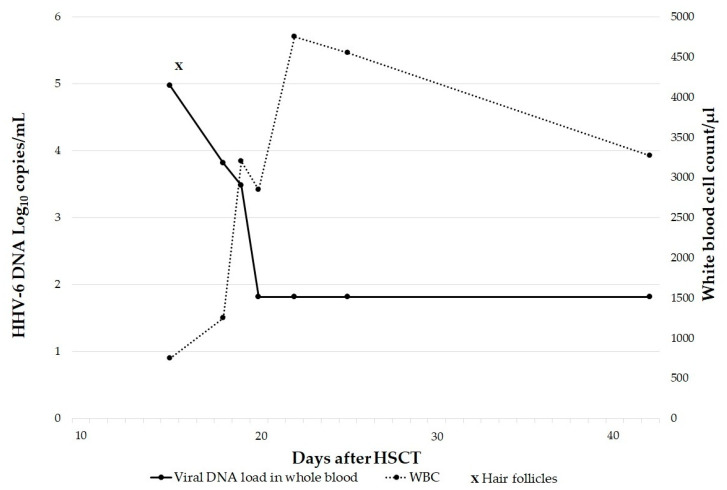
Monitoring of blood HHV-6 DNA post-hematopoietic stem cell transplantation (HSCT) in a patient with iciHHV-6 who received a graft from a donor without iciHHV-6. HHV-6 DNA was related to white blood cell (WBC) count in the early period after transplantation.

Two patients were transplant recipients with transient leukopenia at the moment of blood collection. One was a multivisceral transplant recipient (stomach, duodenum, pancreas, intestine and liver) with HHV-6 DNAemia of 5.9 Log_10_ copies/mL and leukopenia (leukocytes: 710/µL) during routine blood tests. The integration was confirmed by the positive result of the hair follicles test and the subsequent increase of the DNAemia (>6 Log_10_ copies/mL) 1 month after, in correspondence with the leukocyte recovery (leukocytes: 7590/µL). The other patient was an allogeneic hematopoietic stem cell recipient affected by acute myeloid leukemia. He had a facial rash related to GvHD 2 weeks after the transplant and a HHV-6 load in blood at 5 Log_10_ copies/mL (donor-derived leukocytes: 750/µL; chimerism test: 100% donor) with positive hair follicles. The patient was successfully treated with corticosteroids and the viral load quickly decreased without antiviral treatment, confirming the absence of both HHV-6 reactivation in the recipient and iciHHV-6 status in the donor. A persistent HHV-6 low level was detectable in blood after the resolution of GvHD (Figure 1).

Overall, of the 2019 subjects analyzed, 1226 males (61%) and 793 females (39%), 53 were identified as iciHHV-6, indicating a prevalence of 2.6% reaching 9.5% (53/555) considering only the patients with a HHV-6 positive result in blood.

Among the 53 iciHHV-6 patients, 33 were males (62%) and 20 (38%) were females. The sex distribution of iciHHV-6 negative and iciHHV-6 positive patients was similar (*p* = 0.88).

iciHHV-6 cases were divided in 2 age groups: <18 years (16 patients) and ≥18 years (37 patients).

Clinical data from the 53 iciHHV-6 patients are summarized in Table 2 and Table 3. For each patient, we describe: clinical features that required HHV-6 investigation; viral load in blood and, when requested, in cerebrospinal fluid (CSF); patient’s diseases including those already known in medical history and those diagnosed during the current hospitalization. In particular, diseases were classified in 5 groups: hematological, autoimmune, autoimmune neurological, not-autoimmune neurological and other disease (a disease not included in the previous disease categories). Patients without already known pathologies in anamnesis at the hospital admission and without new clinically relevant diseases diagnosed during the hospitalization, were defined as patients without underlying medical conditions (nUMC).

In 23 patients (43%), HHV-6 DNAemia was performed after a HHV-6 positive result obtained in CSF collected to investigate an infectious or neurological or autoimmune disease. The clinical feature leading to HHV-6 DNA investigation was mainly the occurrence of fever, especially in pediatric patients (12/16, 75%).

No iciHHV-6 patients reported angina at the moment of HHV-6 investigation or in the medical history.

## 4. Discussion

Like other herpesviruses, HHV-6 establishes latency following primary infection, allowing the virus to persist in the host for life. In contrast to other human herpesviruses, HHV-6 can integrate its genome into the telomere region of host chromosomes of latently infected cells [22,41].

The HHV-6 integration process is facilitated by telomeric repeats present at the ends of the viral DNA [26] and occurs in a small proportion of somatic cells [7], including monocytes, macrophages, T-cell, or bone marrow progenitor cells [24,25]. However, if integration occurs in the chromosomes of germ cells, vertical transmission of integrated HHV-6 results in offspring with an integrated copy of the virus in each nucleated cell, a phenomenon referred as iciHHV-6 [11,21]. These individuals will transmit the condition to half of their offspring via Mendelian mechanism and exhibit a persistent high viral load in whole blood. Pellet et al. [5] reported that iciHHV-6 individuals have at least one copy of viral genome per white blood cell, which corresponds to >5.5 Log_10_ copies/mL of whole blood. Thus, HHV-6 levels > 5.5 Log_10_ copies/mL in whole blood are suggestive of iciHHV-6 and confirmation can be made by DNA PCR testing of blood-free samples such as hair follicles or fingernails [34,42].

Other authors [3,7] reported that iciHHV-6 individuals can be identified by their constant viral load of 6–7 Log_10_ copies/mL in whole blood. However, when the white blood cell count is significantly decreased, as observed in transplant recipients, the results for absolute viral loads in blood may be ambiguous [6,39].

In the last ten years, we investigated HHV-6 DNAemia in 2019 patients obtaining a positive result in 555 (27.5%). In order to investigate iciHHV-6 status, we used a cut-off of 5 Log_10_ copies/mL in whole blood, lower than the one usually reported (5.5), considering that transplant recipients with HHV-6 reactivation and children with primary HHV-6 infection have transient virus DNA loads typically not more than 5.0 Log_10_ copies/mL in whole blood [5].

In all 59 (2.9%) patients with HHV-6 load greater than or equal to 5 Log_10_ copies/mL in blood, the HHV-6 PCR test on hair follicles was used to confirm or exclude iciHHV-6 status.

We observed that in the 49 patients with HHV-6 DNAemia exceeding 6 Log_10_ copies/mL (range: 6.0–7.2), viral DNA was also detected in hair follicles, confirming iciHHV-6 status.

In the 10 patients with a viral load between 5 and 5.9 Log_10_ copies/mL, hair follicles assay was negative in 6 of them, all infants. They were under 2 years of age with clinical features that required admission to hospital and had a transient viral load between 5.1 and 5.5 Log_10_ copies/mL, higher than the value described by other authors in case of primary infection [5,40]. These results indicate that viral loads corresponding to active infections are located within a wide range, reaching several hundreds of thousands of copies per mL of blood in infants with primary infection. These values may be confused with those resulting from iciHHV-6 [5].

In the remaining 4 patients with a viral load between 5 and 5.9 Log_10_ copies/mL, all with leukopenia at the moment of HHV-6 blood investigation, the suspected iciHHV-6 status was confirmed by the hair follicles’ positive PCR. It is known that in individuals with iciHHV-6, the HHV-6 DNA load in blood varies according to the number of cells included in the specimen, especially when the patient has leukopenia [5,43]. At the moment of blood collection, 2 patients had an acute hematologic malignancy, one was a multivisceral transplant recipient and one was an HSCT.

In HSCTs there are two different scenarios [44,45]: (i) HSCT recipients receiving a hematopoietic cell graft from iciHHV-6 positive donors will have HHV-6 DNA in all donor hematopoietic cells following engraftment [46,47,48]; (ii) HSCT recipients who independently have iciHHV-6 will retain iciHHV-6 in their non-hematopoietic cells after transplant [49]. We observed the second scenario, in particular our iciHHV-6 HSCT patient (Figure 1) developed GvHD 2 weeks after the transplant alongside with a viral load of 5 Log_10_ copies/mL. He was treated with corticosteroids and blood viral load quickly decreased, excluding HHV-6 reactivation. It is known that HSCT patients, particularly those receiving allogeneic transplants, are at high risk of developing a reactivation within the first 4 weeks after cell transfer [6]. The clinical symptoms associated with HHV-6 reactivations are included in a wide spectrum of syndromes: some symptoms may be considered nonspecific, such as fever, and rash, while subacute limbic encephalitis and delayed engraftment are recognized as typical opportunistic diseases due to HHV-6 reactivation in HSCT recipients [6,12,50]. In our case we also excluded iciHHV-6 in the donor because of the extremely low HHV-6 DNA loads after the transplant. Clark et al. [46] have reported the transmission of iciHHV-6 via hematopoietic stem cells from an iciHHV-6 donor to a recipient originally without viral integration, with a consequent enormous increase in the amount of HHV-6 DNA in recipient’s blood. Here, we reported the opposite donor–recipient situation: a recipient with iciHHV-6 who received a graft from a donor without iciHHV-6. Therefore, because the virus remains in the non-hematopoietic cells in the iciHHV-6 recipient’s body, the HHV-6 DNA in blood after transplantation was most likely due to the release of chromosomal DNA from non-hematopoietic cells [49]. During inflammation and GvHD this phenomenon is more evident and the viral load increases [49]. It was also observed in our iciHHV-6 patient affected by acute myeloid leukemia, who developed hepatic and intestinal GvHD after HSCT associated with HHV-6 load of 3.4 Log_10_ copies/mL. Hill et al. [51] reported that acute GvHD was more frequent when recipients or donors had iciHHV-6 and suggested that screening iciHHV-6 could guide donor selection, risk stratification and treatment strategies post-HCT, but further studies are needed.

The results of this study indicate that the prevalence of iciHHV-6 in our population of hospitalized patients is 2.6% (95%CI 2.0–3.4), falling within the 0.2–2.9% range previously reported, depending on the population and region investigated [28,30,31,32,33]. In the literature, in healthy blood donors it was found that 0.8% (4/500) [31] and 1.5% (10/653) [52] had high HHV-6 loads most likely attributed to integration in the germline. In contrast, prevalence of iciHHV-6 in hospitalized patients was higher, 2.9% (13/449) [31] and 3.3% (6/184) [52], respectively. Moreover, we observed that the prevalence of iciHHV-6 did not differ in males and females, similarly to the sex distribution of iciHHV-6 negative and iciHHV-6 positive patients reported in a large scale study [9].

Considering the higher incidence in hospitalized individuals, some authors suggested that iciHHV-6 represents a risk factor for the development of certain medical conditions [5,9].

Recently, the first large scale analysis of disease association with iciHHV-6 has shown that iciHHV-6 individuals have three times greater risk of developing angina pectoris. Authors analyzed DNA from 19,597 participants aged 40–69 years for the identification of iciHHV-6 and determined the telomere length in randomly selected samples. They observed that iciHHV-6 individuals have shorter telomeres than aged-matched control subjects, a result that may partially explain how iciHHV-6 may contribute to the development of angina. Their results support literature data linking short telomere lengths and cardiovascular disease development [9]. In our study, the lack of angina at the moment of HHV-6 investigation or in the medical history could be correlated with the small number of iciHHV-6 positive patients.

HHV-6 integration is not site-specific and can potentially occur in the telomere region of any host chromosome, either the long or short arms [39,53]. To date, iciHHV-6 has been identified in a number of chromosome including 1q, 6q, 7q, 9q, 10q, 11p, 17p, 18p, 18q, 19q, 22q, and Xp [11,21,27,28,29,41,46,54,55,56,57,58,59,60,61].

Integration of HHV-6 within that telomeric region is unlikely to be without clinical consequences [9,19]. Since integration can occur in different chromosomes, the potential pathologies associated with this condition are likely to vary greatly [9,62]. Huang et al. [63] reported that human telomeres carrying integrated HHV-6 are often shorter and unstable. Indeed, it is known that telomeres are the main protectors of chromosomes, and their integrity could be compromised by a viral insertion at this site: the long-term consequence of a shortened telomere could cause premature senescence or cell apoptosis when numerous cell divisions are required [9,62,64]. Several diseases are linked with telomere dysfunctions and/or telomerase mutations such as cardiovascular diseases, hematopoietic dysfunction, pulmonary fibrosis, liver disease, degenerative diseases, and cancer [65,66,67,68,69,70,71,72,73,74,75]. Alterations within telomeric regions are therefore a likely cause for cellular dysfunctions linked to diseases [9,21].

More recently, Wight et al. [76] developed a genome imaging approach, which revealed, contrary to what was previously thought, the telomere lengths of chromosomes harboring the integrated virus genome were comparable to other chromosomes. Therefore, this feature needs further investigations.

In addition, several researchers reported an increase in anti-HHV-6 antibody titers as well as HHV-6 DNA copies while monitoring clinical relapse of multiple sclerosis in patients compared to control subjects [20]. However, pertaining to iciHHV-6, the clinical link between neurological disease and viral replication is still being explored.

In the present study we investigated the occurrence of hematological, autoimmune, autoimmune and not-autoimmune neurological and other diseases in 53 iciHHV-6 patients. All of them had HHV-6 load > 5 Log_10_ copies/mL in whole blood and we had performed this evaluation mainly for the occurrence of fever, especially in pediatric patients (12/16, 75%), or of symptoms requiring lumbar puncture to investigate an infectious or neurological or autoimmune disease.

Patients without a diagnosis of a specific pathology in anamnesis and at hospital discharge were defined nUMC patients and, as expected, the frequency was significantly higher in pediatric patients (9/16) versus adults (2/37) (*p* = 0.002). Focusing our attention on the 37 adult iciHHV-6 patients, we observed in 41% of cases a neurological disease and in 24% and 11% a hematological and autoimmune disease, respectively. All the 9 adult hematological patients had a blood tumor and 3 of them also had a solid tumor. Overall, 13/37 (35%) adults were affected by neoplasia. Nevertheless, the small number of iciHHV-6 positive patients and the condition of hospitalization do not permit us to achieve a correlation between HHV-6 integration and specific pathologies. In addition, given the retrospective nature of our study, we do not have information regarding the medical condition in patients without iciHHV6. Further prospective studies that evaluate the prevalence of iciHHV-6 positive and negative status in specific medical conditions are needed.

Although there is no evidence linking iciHHV-6 to any cancer type, some authors suggested that iciHHV-6 might predispose the formation of marker chromosomes [77]. In the case of a patient with diffuse large B-cell lymphoma (DLBCL) and iciHHV-6 integration in chromosome 17p the authors pointed out that structurally abnormal chromosomes are commonly found in DLBCL, and that the high expression of HHV-6 U94, a protein with DNA-binding, exonuclease, and helicase-ATP activities, may have been involved in tumorigenesis [77,78].

Our study presents the following limitations: first, the high frequency of neurological and autoimmune diseases could be correlated with the high percentage of patients (43%) in whom HHV-6 DNAemia was performed after a positive result in CSF collected to evaluate an infectious or neurological or autoimmune disease; and second, the clinical data and laboratory findings regarding iciHHV-6 negative patients were not available.

We observed that HHV-6 DNAemia values more than 6.0 Log_10_ copies/mL were always correlated with iciHHV-6. In case of viral loads between 5.0 to 5.9 Log_10_ copies/mL, we identified the following patients’ categories: iciHHV-6 positive leukopenic patients; iciHHV-6 positive patients receiving HSCT from donors without integration; iciHHV-6 negative pediatric patients with primary HHV-6 infection. Therefore, attention should be paid to avoid erroneous diagnosis of active infection and unnecessary treatment.

The prevalence of iciHHV-6 in our hospitalized patients was 2.6%, higher than what reported in literature in blood donors (0.8–1.5%), suggesting that iciHHV-6 represents a risk factor for the development of certain medical conditions.

In our study population, 95% of iciHHV-6 adult hospitalized patients presented a wide range of pathologies. The current understanding of clinical consequences of iciHHV-6 is only in its initial stage of development. By altering the telomeric regions, HHV-6 may affect the ability of cells to divide, leading to organ dysfunctions or may provoke chromosomal instability. Additional large-scale clinical-epidemiological studies are required to explore association between the specific HHV-6 integration sites and the occurrence of specific diseases.

## Figures and Tables

**Table 1:** Identification of iciHHV-6 status among the 59 patients with a viral load in blood ≥ 5.0 Log_10_ copies/mL.

HHV-6 DNA Log_10_ Copies/mL Whole Blood		No. of Patients	HHV-6 DNA Hair Follicles
≥6.0		49	positive
5.0–5.9	(5.1–5.5)	6 ^a^	negative
	(5.0–5.2)	2 ^b^	positive
	(5.0–5.9)	2 ^c^	positive

^a^: infants with acute HHV-6 infection; ^b^: patients with hematologic malignancy; ^c^: transplant recipients.

**Table 2:** Overview of the 37 iciHHV-6 adult patients: clinical features leading to HHV-6 investigation, HHV-6 DNA load in CSF and WB and patients’ disease.

Case	Sex	Age (y)	Clinical Features	CSF (Log_10_ Copies/mL)	WB (Log_10_ Copies/mL)	Category	Disease
1	M	74	Fever	/	6.0	H	Chronic lymphocytic leukemia and diffuse large B-cell non-Hodgkin lymphoma Other: prostate cancer
2	M	70	Screening	/	6.4	H	B-cell non-Hodgkin lymphoma Other: hypertensive heart disease
3	M	67	Fever, erythema	/	5.0	H	Acute myeloid leukemia Other: allergy to cephalosporins; bladder neoplasia; metasteroid diabetes mellitus; gastrointestinal and hepatic GvHD; hypertension
4	F	48	Facial rash (GvHD)	/	5.0	H	Acute myeloid leukemia; allogeneic transplant Other: papillary thyroid carcinoma
5	M	46	Screening	/	6.1	H	Sezary syndrome
6	M	46	Fever, hematemesis, melena	/	6.2	H	Castelman’s disease Other: HIV infection
7	F	42	Fever, headache	5.3	6.3	H	Hodgkin’s lymphoma Other: infertility; pericarditis; enterovirus meningitis
8	M	33	Fever, rash	/	6.4	H	Acute myeloid leukemia; allogeneic transplant Other: Gilbert’s syndrome
9	M	18	Screening in chemotherapy	/	5.2	H	Acute lymphoblastic leukemia
10	F	41	Worsening subacute medullary syndrome	3.1	6.7	A	Graves’ disease Other: inflammatory cervical spinal cord injury
11	M	37	Fever, confusional state	4.4	6.4	A	Autoimmune thrombocytopenia Other: HIV infection; allergy to acetylcysteine
12	F	36	Acute renal failure, severe anemia	/	7.2	A	Hashimoto’s thyroiditis; Goodpasture’s syndrome
13	M	31	Limbs weakness, dysesthesia	3.4	6.5	A	Rheumatoid arthritis
14	F	56	Diplopia, ataxia, asthenia, tetraparesis	/	6.6	N(A)	Axonal and demyelinating polyradiculoneuropathy
15	F	49	Dysesthesia and limbs weakness	3.6	6.1	N(A)	Multiple sclerosis Other: Hay–Wells syndrome
16	F	46	Weakness, paraesthesia in limbs	4.8	6.5	N(A)	Acute disseminated rncephalomyelitis Other: allergy to penicillin, cephalosporins, amoxicillin
17	M	33	Diplopia, ataxia	3.0	6.4	N(A)	Inflammatory demyelinating disease of the CNS
18	F	30	Dysesthesia in lower limbs	/	6.5	N(A)	Multiple sclerosis
19	M	28	Dysarthria, nuchal rigidity	3.2	6.7	N(A)	CNS demyelinating disease
20	M	20	Left hemiplegia	3.3	6.7	N(A)	CNS demyelinating disease
21	F	84	Fever, sleepy state	3.8	6.7	N(nA)	Parkinsonism Other: chronic ischemic heart disease; hypertension
22	F	81	Weakness of lower limbs in suspected polyradiculoneuritis	3.3	6.4	N(nA)	Encephalopathy and peripheral neuropathy
23	M	59	Confusional state	3.3	6.7	N(nA)	Cerebral ischemia and microbleeds Other: insulin dependent diabetes mellitus; hypertension
24	M	54	Dengue infection	/	6.0	N(nA)	Bell’s palsy Other: metabolic syndrome; prostatic hyperplasia
25	M	51	Sensitive limbs disorders	2.9	6.3	N(nA)	History of neurally-mediated sincopes
26	F	40	Fever, seizures	/	6.5	N(nA)	Super refractory cryptogenic status epilepticus Other: primary immune regulatory disorder; sclerosing cholangitis; ovarian cancer; skin psoriasis; allergy to phenobarbital, phenytoin, augmentin
27	M	38	Worsening of respiratory dynamics	/	6.6	N(nA)	Amyotrophic lateral sclerosis Other: history of febrile seizures
28	F	20	Decreased vision, headache	3.1	7.2	N(nA)	Idiopathic intracranial hypertension
29	M	61	Pneumonia (HHV-6 positive bronchoalveolar lavage)	/	6.5	O	Liver transplant for cirrhosis
30	M	58	Fever, headache	/	6.6	O	Liver transplant for hepatocellular carcinoma
31	M	44	Heart failure	/	6.5	O	Dilated cardiomiopathy
32	F	40	Screening in transplant	/	5.9	O	Multivisceral transplant for small bowel syndrome; resistance to ganciclovir
33	F	39	Jaundice	/	6.3	O	Liver transplant for type IV Klatskin tumor
34	M	29	Screening	/	6.1	O	Kidney transplant for renal hypoplasia
35	M	24	Syncope, chest pain	/	6.0	O	Ewing’s sarcoma with pulmonary metastasis
36	F	84	Confusional state, auditory and visual hallucinations	3.0	6.7	nUMC	
37	M	37	Fever, headache	5.8	6.8	nUMC	

GvHD—graft-versus-host-disease; CSF—cerebrospinal fluid; /—not performed; WB—whole blood; H—hematological; A—autoimmune; N(A)—autoimmune neurological; N(nA)—not-autoimmune neurological; O—other; nUMC—no underlying medical disease; CNS—central nervous system.

**Table 3:** Overview of the 16 iciHHV-6 pediatric patients: clinical features leading to HHV-6 investigation, HHV-6 DNA load in CSF and WB and patients’ disease.

Case	Sex	Age (y)	Clinical Features	CSF (Log_10_ Copies/mL)	WB (Log_10_ Copies/mL)	Category	Disease
1	M	8	Screening for bone marrow transplant	/	6.4	H	Myd88 deficiency
2	F	16	Fever, headache, syncopal episodes	4.0	6.6	A	Henoch–Schönlein purpura Other: ostium secundum atrial septal defect
3	F	0	Fever, vomiting	3.0	6.5	A	Kawasaki disease with coronary aneurysms
4	M	11	Fever, soporous state	3.3	6.6	N(nA)	Syndrome of transient headache and neurologic deficits with CSF lymphocytosis
5	M	7	Fever, hemolytic uremic syndrome	/	6.8	N(nA)	Posterior reversible encephalopathy syndrome
6	M	1	Fever, seizures	2.7	6.9	O	Death from unknown causes Meningitis
7	M	0	Fever, tremors	/	6.9	O	Ventricular septal defect
8	M	15	Fever, asthenia	/	6.4	nUMC	
9	F	15	Fever	3.1	6.3	nUMC	
10	M	8	Bone marrow donor to his twin brother	/	6.4	nUMC	
11	M	4	Rash	/	6.6	nUMC	
12	M	1	Fever, seizures, acute Epstein-Barr virus infection	/	6.5	nUMC	
13	F	0	Fever, jaundice	/	6.2	nUMC	
14	M	0	Fever, rash	/	6.7	nUMC	
15	F	0	Fever	3.0	6.6	nUMC	
16	M	0	Dyspnea	5.6	6.1	nUMC	

CSF—cerebrospinal fluid; /—not performed; WB—whole blood; H—hematological; A—autoimmune; N(nA)—not-autoimmune neurological; O—other; nUMC—no underlying medical disease.

## Data Availability

All the data used in this study are available from the corresponding author upon request.
